# Fostering participation of general practitioners in integrated health services networks: incentives, barriers, and guidelines

**DOI:** 10.1186/1472-6963-9-48

**Published:** 2009-03-17

**Authors:** Matthieu de Stampa, Isabelle Vedel, Howard Bergman, Jean-Luc Novella, Liette Lapointe

**Affiliations:** 1 University of Versailles St-Quentin, Santé Vieillissement Laboratory, AP-HP, Sainte Perine Hospital, Paris, France; 2 Solidage Research Group on frail older persons, McGill – University of Montreal, Montreal, Quebec, Canada; 3 Division of Geriatric Medicine, McGill University, Jewish General Hospital, Montreal, Quebec, Canada; 4 University of Reims Champagne Ardennes, Santé Publique, Vieillissement et troubles cognitifs et du comportement Laboratory, Sébastopol Hospital, Reims, France; 5 Desautels Faculty of Management, McGill University, Montreal, Quebec, Canada

## Abstract

**Background:**

While the active participation of general practitioners (GPs) in integrated health services networks (IHSNs) plays a critical role in their success, little is known about the incentives and barriers to their actual participation.

**Methods:**

Data were gathered through semi-structured interviews and a mail survey with GPs enrolled in SIPA (system of integrated care for older persons) at 2 sites in Montreal. A total of 61 GPs completed the questionnaire, from which 22 were randomly selected for the qualitative study, with active and non-active participation in the IHSN.

**Results:**

The key themes associated with GP participation were clinician characteristics, consequences perceived at the outset, the SIPA implementation process, relationships with the SIPA team and professional consequences. The incentive factors reported were collaborative practices, high rates of elderly and SIPA patients in their clienteles, concerns about SIPA, the selection of frail elderly patients, close relationships with the case manager, the perceived efficacy of SIPA, and improved professional practices. Barriers to GP participation included high expectations, GP recruitment, lack of information on SIPA, difficult relationships with SIPA geriatricians and deterioration of physician-patient relationships. Four profiles of participation were identified: 2 groups of participants active in SIPA and 2 groups of participants not active in SIPA. The active GPs were familiar with collaborative practices, had higher IHSN patient rates, expressed more concerns than expectations, reported satisfactory relationships with case managers and perceived the efficacy of SIPA. Both active and non-active GPs reported quality care in the IHSN and improved professional practice.

**Conclusion:**

Throughout the implementation process, the participation of GPs in an IHSN depends on numerous professional (clinician characteristics) and organizational factors (GP recruitment, relationships with case managers). Our study provides guiding principles for establishing future integrated models of care. It suggests practical guidelines to support the active participation of GPs in these networks such as physicians with collaborative practices, recruitment of significant number of patients per physicians, the information provided and the accompaniment by geriatricians.

## Background

All across the industrialized world, programs have been developed to deliver integrated services in response to the double challenge posed by aging populations and the fragmentation of health services. Frail elderly persons present acute and chronic medical, physiological and social problems[1] and run a higher risk of hospitalization and institutionalization[2,3]. It has been shown that these complex needs require a complex combination of community-based health and social services in long-term care[4]. Furthermore, the fragmentation of health service delivery is particularly problematic for the frail elderly as it results in discontinuity and poorer quality of care[5]. In response to these challenges, public policies have been increasingly directed at coordinating actions and improving public health by integrating services with long-term case management[6,7].

Integrated health service networks (IHSNs) have been developed to improve continuity and increase the efficacy and efficiency of services, especially for older and disabled populations. Kodner and Kyriacou define integrated care as "a discrete set of techniques and organisational models designed to create connectivity, alignment and collaboration within and between the cure and care sectors at the funding, administrative and/or provider"[8]. In these models, the focus is on patients' needs and is provided by an interdisciplinary team that includes the case manager and the patient's general practitioner (GP). The case manager is usually but not always a nurse who identifies patients suitable for the program and collaborates with the team to coordinate care and prevent deteriorating health conditions[9,10]. Recent evaluations of integrated health care models in primary care have yielded promising results and have shown that it is possible to reorganize care, achieving better overall quality and improving satisfaction levels without increasing system costs[11,12].

The active participation of general practitioners (GPs) in IHSNs plays a critical role in their success by enhancing their effectiveness. GPs, who are usually the gatekeepers of the health system, constitute the pivotal medical practitioner and coordinate the specialized medical services[13]. It would appear that medical participation in the network increases patient adherence[14] and that, without it, patient adherence diminishes[15]. Moreover, it seems that the inability of interventions to demonstrate significant improvements in quality of care could be explained by difficulties faced by the network team, to mobilize the community medical intervention[12,16]. The active participation of GPs in IHSNs seems, however, limited[17], despite the fact that recent research has shown that in health care systems, policy implementation depends upon provider commitment[18]. Nonetheless, in research and practice, very little attention has been paid to GP participation in IHSNs.

The goal of our study was to fill this gap by identifying incentives and barriers associated with medical active and non-active participation in an IHSN for frail older persons. To this end, we examined the participation of physicians registered in a Québec experiment called SIPA (the French acronym for System of Integrated Care for Older Persons), an integrated model for delivering care to the frail elderly tested at 2 sites in Montreal. Given the vital role played by GPs, our study provides practical guidelines for both research and practice and may contribute to more effective management of services through public policy initiatives.

## Methods

The active participation of GPs in IHSNs is a complex phenomenon that depends on numerous factors. After conducting a short survey, we decided to use qualitative methods to develop a deeper understanding of the dynamics of IHSN implementations. All the GPs interviewed in the present study were registered in networks. We were studying participation in a longitudinal retrospective perspective. In this study, registration of GPs refers to the GP's initial decision to participate in the IHSN. Our focus, however, is on their "ongoing participation," which refers to continuous collaboration and active involvement over time, thereby distinguishing active participants from non-active participants.

### Description of the Integrated Health Services Network

In the late 1990s, Solidage–a joint University of Montreal/McGill University research group on integrated services for older persons–designed SIPA (system of integrated care for older persons), a model aimed at comprehensive, integrated health and social service interventions for frail elderly populations. SIPA is a community-based, patient-focused, on the needs of the frail elderly population in a given territory of Montreal, Québec. The target population was individuals with severe disabilities (65 years of age or older, community-dwelling, residing in one of the 2 sites and being frail) as assessed by the functional autonomy measurement system (SMAF) scale, which includes activities of daily living (ADL), instrumental ADL (IADL), communication, and cognition[19]. SIPA had the specific objectives of ensuring comprehensive, continuous care and mobilizing a needs-based, flexible, and rapid response. SIPA was responsible for the delivery of all services, including health and social services and acute and long-term community and institutional care (acute care hospitals and nursing homes). Case managers intervened in medical and social issues, interacting with patients and caregivers and consulting with the attending general practitioner. A series of evidence-based interdisciplinary protocols (on nutrition, falls, congestive heart failure, dementia, depression, medication, vaccination) were established. GPs were the main medical actors, and they were expected to provide home care, share information with the case managers and follow SIPA guidelines. They were paid $400 per SIPA patient annually, in addition to their usual fee-for-service payments, to compensate for the time spent communicating with the team. The SIPA team was accountable for service utilization in terms of hospital stays as well as services provided in the community; its members monitored the application of protocols[20].

The SIPA model was applied in a project carried out over a 22-month period from June 1999 to March 2001 at two sites in Montreal community health centres (CLSCs, the public community-based organizations responsible for home care in the province of Québec). A site was defined as the setting of the model implementation. Site 1 was located in the north east of the Montreal Island, with a French speaking population and a household above-average income. Site 2 comprises a multiethnic, mainly English-speaking population in the western part of the Island, with dramatic contrasts in socio-economic levels. A detailed description of each site is provided in appendix. By using these 2 sites, we were able to compare "homogeneous" samples in situ. At each site, the frail elderly were selected first and then their GPs were asked to participate in the program. Of the 113 registered GPs (who agreed to collaborate and receive annual stipend), 31 became active participants in the experiment for the 22 months of the study (27.4% of the sample). The participation of GPs in SIPA was assessed by 3 independent professionals (2 case managers and 1 manager of care) based on 2 items: answering the case manager's phone calls and treating frail patients in less than 48 hours. GPs were considered active participants when they met the two conditions; otherwise, they were considered non-active participants. The vast majority of the non-active participants did not fulfill any of their commitments. The inter-rater agreement was 0.80, and very few GP cases had to be discussed by the 3 professionals in order to reach a consensus.

### Study population

All the 113 registered GPs were sent a mail-in questionnaire. Among physicians who completed this survey, we randomly selected 22 GPs on a pro-rata basis according to the participation level – making sure that we were selecting active and non-active GPs – at the two sites. Indeed, we purposely included physicians from the two groups. Each of these randomly selected physicians received a letter from two senior SIPA physicians explaining the study's objectives. They were then contacted by telephone to set up an interview. To encourage a positive response, physicians were offered $100/interview. Four GPs refused to participate, so more names were drawn. No differences were noted between the profiles of our respondents and the profiles of those who refused to participate. As per the Sir Mortimer B. David – Jewish General Hospital ethics committee research guidelines, all the study participants were fully informed of the research objectives and were asked to provide written consent before the beginning of the interviews.

### Data collection

This study used a mixed method with quantitative and qualitative findings. First, quantitative data were collected by administering a short mail survey that provided socio-demographic information, data on the type of practice (number of years, general practitioner or specialist, number of elderly patients) and the number of elderly patients selected for the SIPA experiment. Qualitative data came from semi-structured, face-to-face interviews. All interviews were conducted by the same person, a physician who was trained in qualitative research and who is one of the study's investigators but who was not involved in the SIPA trial. The interviews were held in each physician's place of practice. All the interviews lasted from 45 to 60 minutes. The design of the questionnaire was based on a review of the literature[21,22] and was validated in the field with two test interviews in order to develop the content and the process. The interviews began with a very general question concerning professional habits and then moved on to more specific questions exploring three themes: 1) their motivation for registering with and participating in SIPA, 2) specific aspects of the SIPA model and its implementation, and 3) SIPA's impact on their professional activities. A preliminary categorization of incentives and barriers took place following the first 6 interviews. Saturation emerged after 16 interviews and the remaining data were used to validate the categories.

### Coding and analysis

Interviews were recorded in their entirety and transcribed verbatim. Through coding and analysis, we identified relevant categories and relationships. NVivo6 (software for qualitative data analysis) was used for data coding, which was validated by three researchers. Afterwards, tables were created to compare active and non-active participants. A number of excerpts from the interview transcripts highlighted specific behaviors; these excerpts were organized in conceptually clustered matrices so as to build a logical chain of evidence to develop an explanation-building analytic strategy[23]. We then performed a cross-case analysis to identify common patterns and unique features at the 2 sites. This second analysis captured new elements that added to our understanding and also highlighted emergent patterns. The analytical process was repeated until saturation (the point at which additional data repeatedly confirm the interpretation already made).

### Ethic certificate and delayed publication of the results

The study was approved by the Research Ethics Committee of the Sir Mortimer B. David – Jewish General Hospital (#98-075). Though the SIPA experience began in 2001, the empirical evidence reported in this article was re-examined only recently. Indeed, following the implementation of a similar model of care (the COPA model in Paris) by the principal investigator of this study, it became clear that the question of GPs' participation is a critical issue in the success of the implementation of these integrated networks. Therefore, it was deemed important to revisit the data gathered about the SIPA experience in order to share its lessons with the medical and scientific communities. Data were thus re-analyzed in 2007, which explains the publication delay.

## Results

A total of 61 registered GPs completed the mail-in questionnaire (54% response rate after one recall), and 22 of these GPs were interviewed at the 2 sites: 9 GPs at Site 1 and 13 GPs at Site 2. Among the 22 interviewees, 10 were active participants and 12 were non-active. We grouped the results of the data analysis under five emergent key themes of incentives and barriers to GP participation in an IHSN (see Table 1).

**Table 1:** Incentives and barriers to GPs participation in IHSNs

	**Incentives**	**Barriers**
**Clinician characteristics**	- Having elderly persons in clientele - Having a high number of SIPA patients **- **Having collaborative practices	
		
**Perceived consequences**	- Initial concerns	- Initial expectations
		
**IHSN implementation**	- Selection of frail elderly patients - Influence of peers	- Short-term duration - Physicians recruited after patients - Little information provided
		
**Relationship with SIPA team**	- Close relationships with case managers	- Conflict with the geriatrician - Lack of communication on patients
		
**Professional consequences**	- Efficacy of SIPA - Quality of SIPA - Funding - Improved professional practice	- Physician-patient relationships

### Clinician characteristics

Clinician characteristics were compared between the survey and the interviews; all GPs were similar. The majority of the GPs were male, English-speaking and general practitioners with long-term professional practices. They had low rates of elderly patients among their clients, were unfamiliar with collaborative practices and had fewer than 3 frail elderly patients selected for SIPA (2.7 for the survey group and 2.8 for the interview group). The active interviewees (as compared to non-active) were general practitioners, most were French-speaking and no difference was found in term of number of years of practice. Active interviewees had a higher rate of elderly patients in their clienteles, were familiar with collaborative practices and had more frail elderly persons selected for SIPA as compared to the non active respondents (4.6 versus 1.3, respectively) (see Table 2).

**Table 2:** Characteristics of GPs in the survey and in interviews

	**Survey** **(n = 61)**	**Total** **Interviewees** **(n = 22)**	**Active** **(n = 10)**	**Non Active** **(n = 12)**
**Sex – male %(n)**	67.2 (41)	63.6 (14)	60 (6)	66.6 (8)
				
**Mother tongue**				
**%(n)**				
**French**	32.8 (20)	36.4 (8)	60 (6)	16.8 (2)
**English**	52.5 (32)	50 (11)	30 (3)	66.6 (8)
**Other**	14.7 (9)	13.6 (3)	10 (1)	16.6 (2)
				
**General practitioners % (n)**	85.2 (52)	86.4 (19)	100 (10)	75 (9)
				
**Years of practice (average)**	28.3	28.2	28.7	27.8
				
**Rate of elderly patients in clientele**				
**< 25%**	50.8 (31)	54.6 (12)	0	100 (12)
**25–50%**	27.9 (17)	27.2 (6)	60 (6)	0
**> 50%**	21.3 (13)	18.2 (4)	40 (4)	0
				
**Collaborate with home care team (yes) % (n)**	31.2 (19)	36.4 (8)	60 (6)	16.6 (2)
				
**Number of frail elderly patients registered with SIPA**	2.7	2.8	4.6	1.3
				
**Active GPs %**	42.6	45	100	0

### Perceived consequences at the outset

Interviewed GPs expressed a few initial concerns and some expectations about their participation in this new IHSN at the beginning of SIPA.

#### Initial concerns

Most of the active GPs at the 2 sites indicated that they initially feared that they would not have enough time to fully participate in the experiment. These fears were exacerbated by the fact that the GPs had limited information at the beginning of the program.

*Active GP8: I was afraid that it would require a very large commitment. Would it require that I provide many hours of my time? At the time, I had a very tight schedule that didn't necessarily allow me to see a patient the same day or within a day's notice*.

The non-active GPs were afraid that they would be overburdened by administrative tasks and not benefit from any time they might save.

*Non-active GP4: I didn't know what it would be, if it would be too much time on the telephone, too much paperwork. I was willing to participate if it would make my work easier and save me some time*.

#### Initial expectations

Some of the physicians at both sites were hoping that they would receive assistance in supervising home care for their frail patients. Some of the non-participants expected to share the burden and offload their heavier cases to SIPA.

*Non-active GP11: I didn't want to rely on SIPA but, rather, to share the burden of a patient with SIPA (...) It was something new that met a real need*.

Active physicians at both sites indicated that, early on in the project, they did not have clearly defined expectations as to what could be gained from SIPA. Some of them said that they registered in SIPA in order to maintain relationships with their patients.

*Active GP16*: *By necessity, but not willingly; I didn't want to abandon my patients. (...) As I said, it was out of necessity; I did it for the patient*.

### Implementation of SIPA

GPs criticized the implementation of the program in terms of the recruitment of physicians, its length and communications under the program.

#### Selection of patients

All GPs at both sites were satisfied with the selection of frail elderly patients for SIPA. They agreed with the principle that patients should be selected for admission into an IHSN and that it should identify the patients who stood to benefit the most.

*GP13: At the start, patients had to be selected based on which ones stood to benefit and which ones didn't (...) I fully agreed with the selection, and the project needed frail elderly patients*.

#### Physician recruitment

Most of the non-active GPs at the 2 sites were not comfortable with having been recruited after their elderly patients had been selected. They would have preferred being contacted first and asked to submit a list of patients for SIPA. They felt that they are the professionals best placed to know which patients need to be registered in an IHSN.

*Non-active GP3: The physicians need to be brought on board first, because each one knows his clientele and knows that, sooner or later, the patient will enter a hospital or a care facility*.

#### Length of the program

All the physicians were critical of the fact that the SIPA experiment had lasted only 22 months. Active GPs regretted that the short period of time limited SIPA's positive impact on quality of care.

*Active GP5: It was not long enough to allow us to understand how we can better manage our patients with SIPA*.

The non-active physicians stated that the short length kept them from getting involved in the project.

*Non-active GP10: The fact that I knew it was temporary, that bugged me (...). From the start we thought that it wouldn't last, because there was a lot of waste and things couldn't go on that way*.

#### Information provided

Most of the non-active GPs said that they regretted the lack of information provided at the outset on their specific role in the SIPA experiment. They found the role of the case manager and their relationship somewhat vague.

*Non-active GP4: It was confusing for me at the beginning. And afterwards, too. I didn't receive clear information about what I was supposed to do*.

#### Peers' influence

Some of the non-active physicians at Site 2 indicated that they had registered in part as a show of support for the SIPA research physicians present at the site. Active physicians at the same site indicated that the presence of peers did not have an influence on their decision to register in SIPA.

*Non-active GP21: At the start-up, I think it was because of one of my colleagues, I think it was Dr. X, who approached me to see if I would be interested. That's why*.

### Relationships with the SIPA team

During the implementation of SIPA and thereafter, GPs reported that their relationships with the case manager and the geriatrician were critical factors in their involvement in the program.

#### Relationships with the case manager

Active physicians at both sites reported good relationships with the case managers, who kept them informed about their patients. For these GPs, the case managers were professionals who had good knowledge of the frail elderly. GPs collaborated more when they had more elderly patients selected for the experiment.

*Active GP18: It was easy to work with the case managers. They were nurses from community services who knew the condition of my patients very well. I developed really good relationships with some of them, because they had to call me often for the complex cases*.

Most of the non-active GPs at both sites indicated that they were rarely in touch with the case managers. They would have liked to have met them earlier in the process to improve their collaboration. For these GPs, it was difficult to learn how to work with a new professional just by exchanging phone calls.

*Non-active GP7: It was difficult to work with them because I never met them face-to-face. It was only phone conversations. When you have real human contact, it changes something. But with SIPA, you don't know who you're dealing with. You can't put a face on them*.

#### Relationships with the geriatrician

Some GPs at both sites had conflicts with the SIPA geriatrician during the experiment, with differences between active and non-active GPs. For example, some active GPs indicated that the geriatricians had recommended a change in therapeutic prescriptions without knowing the patient.

*Active GP16: The presence of another physician can be a good thing, but sometimes his advice just isn't appropriate, precisely because he hasn't seen the patient, he hasn't seen this, he hasn't seen that. (...) He gives advice from perhaps too great a distance, and obviously it is not going to be followed very closely in our management of patients*.

Non-active GPs stated that they had not had a close enough working relationship with the geriatrician. They disliked sharing clinical responsibility for a given patient with another physician.

Non-active GP17: At one point, the patient had two physicians, because over time the SIPA physician began to take care of our patients, even though we had been recruited to take care of them and we had been working with them from the start (...)

#### Communications about patients

Some GPs at both sites indicated that they did not receive enough information about their patients once SIPA became involved in their care. GPs wanted to be briefed when patient problems occurred because they were not accustomed to calling SIPA. This was verified when GPs had fewer SIPA patients.

*GP12: Perhaps SIPA didn't have all that much time to give us. Not that we're interested in holding team meetings, but it could have been as little as a short letter or fax, or I could have responded in writing or by phone. They didn't keep me in the loop when it came to issues beyond the patient's problems, but I didn't make an effort to find out, either*.

### Professional consequences

GPs indicated that their participation in SIPA has had some impacts on their professional activities.

#### Efficacy of case management

Active GPs developed a positive view of SIPA's capacity to respond to the demand for services (such as physiotherapy, blood tests, etc.). Over the course of the experiment, GPs appreciated SIPA's availability when they needed a specific health service for their patients.

*Active GP1: The contact was made quickly, the response time was short (...) Compared to the traditional services, things went faster, because we called the person in charge and right away we had what we wanted. I have the impression that it was simply a question of people being more available (...) faster access, etc*.

#### Quality of care

All physicians at both sites reported that SIPA had made it possible to offer quality services. Moreover, they indicated that the elderly people who had participated in the project felt safer throughout the experiment because of the quality of the services provided.

*GP2: People in the project benefited from highly appropriate services delivered very professionally (...) In terms of the quality of care, it's clear that there were improvements, because there was constant contact between the patient and the services*.

#### Funding

Some active GPs felt that SIPA's funding (400$/patient) was an appropriate amount for the time spent with the case manager.

*Active GP8: Payment was justified because I got involved in a program where I could be highly solicited. So, I got paid for the telephone calls or dealing with other problems*.

Other active GPs mentioned that they felt that the remuneration was unfair since they had other frail elderly patients outside SIPA, that they gave these patients the same type of care, and that these patients did not receive the same services.

#### Physician-patient relationships

Non-active GPs at both sites reported that their relationships with patients deteriorated during the program. They indicated having lost patients through the implementation by the SIPA team. The case managers were getting accustomed to working with the geriatrician, and the GPs felt excluded.

*Non-active GP21: I didn't know what was going on with my patient. Perhaps he preferred being cared for by the case manager with the geriatrician, I don't know*.

For the active physicians, relationships with patients did not change during the program.

#### Working conditions

Most of the physicians at both sites felt that SIPA had a positive impact on their conditions of practice. For active GPs, input from the case manager allowed them to anticipate health problems and manage day-to-day complex cases more efficiently and, in the final analysis, it allowed them to save time.

*Active GP20: When we know that a case manager will be giving home care, we can decide to put off our own visit, if it isn't a serious medical problem. I know that the patient is safe; I can decide to visit someone else*.

Non-active GPs were pleased to be relieved of their heavier cases. They appreciated no longer handling these cases on their own, and stated that the arrival of SIPA had improved their working conditions.

*Non-active GP12: It was very good help for me. I couldn't treat these frail patients on my own, and I was confident about transferring them to SIPA because SIPA had high-quality services tailored to this population*.

### Profiles of participants

Based on the analysis, it is possible to identify four participant profiles among the 22 GPs interviewed: two profiles of active registered physicians (Profiles 1 and 3) and two profiles of non-active registered physicians (Profiles 2 and 4). The data revealed that each type of active and non-active GP had different characteristics (see Table 3).

**Table 3:** Participant profiles of GPs in IHSNs

**Profile 1- Active GPs** - *% of elderly in clientele*: > 50% - *Collaborating with home care team*: familiar - *SIPA patient/physician in SIPA*: 5.2 - *Initial concerns: *not having enough time to participate - *Initial expectations*: avoid abandoning their patients - *Physician recruitment*: comfortable - *Information on implementation*: Satisfied - *Contact with the case manager*: close relationship - *Contact with the geriatrician: *conflict about recommendations - *Communications concerning patient: *enough information - *Efficacy and quality of SIPA services*: very satisfied - *Funding*: seen as unfair - *Physician-patient relationships: *satisfied - *Professional practice*: were able to do more work in less time and see improved quality of care	**Profile 2- Non-active GPs** - *% of elderly in clientele*: > 25% - *Collaborating with home care team*: unfamiliar - *SIPA patient/physician in SIPA*: 2.4 - *Initial concerns:* - *Initial expectations*: obtain assistance in managing elderly patients - *Physician recruitment*: unsatisfied; wanted to be consulted before patient selection - *Information on implementation*: shortcomings with respect to task distribution - *Contact with the case manager*: not enough - *Contact with the geriatrician: *dislike sharing clinical responsibility - *Communications concerning patient: *lack of information - *Quality of services*: satisfied - *Funding*: useless because they were not often called upon - *Physician-patient relationships: *unwanted patient loss - *Professional practice*: none
	
**Profile 3- Active GPs** - *% of elderly in clientele*: > 25% - *Collaborating with home care team*: unfamiliar - *SIPA patient/physician in SIPA*: 4.1 - *Initial concerns*: would take too much of their time - *Initial expectations*: none expressed - *Physician recruitment*: satisfied - *Information on implementation: *satisfied - *Contact with the case manager*: satisfied - *Contact with the geriatrician*: no conflict - *Communications on patients*: not receiving enough information on their patients - *Efficacy and quality of SIPA services: *very satisfied - *Funding*: justified, given the need to modify practices and work habits - *Physician-patient relationships*: satisfied - *Professional practice*: improved quality because problems were anticipated by the case manager	**Profile 4- Non active GPs** - *% of elderly in clientele*: < 25% - *Collaborating with home care team*: unfamiliar - *SIPA patient/physician in SIPA*: 1.1 - *Initial concerns*: none expressed - *Initial expectation*: offload heavier cases - *Physician recruitment*: unsatisfied; wanted to participate in the selection process - *Information on implementation: *none expressed - *Contact with the case manager*: rare - *Contact with the geriatrician*: none - *Communications on patients*: none expressed - *Quality of SIPA services*: very satisfied, which reinforced their willingness to transfer patients to SIPA - *Funding*: considered a gift because they did not actively participate - *Physician-patient relationship*: satisfied - *Professional practice: *they were relieved of their heavier cases

***Profile 1 active GPs ***had work habits that matched the SIPA model (type of patients, collaborative practices). They had the highest number of SIPA patients selected from their practices (5.2 per physician on average). They were afraid that they would not have enough time to participate and wanted to avoid abandoning their patients. They were comfortable with how physicians were recruited. They collaborated well with case managers, had enough information on their patients, but had conflicting relationships with the geriatrician. They were satisfied with the efficacy and quality of SIPA services. They considered the funding arrangement unfair. They reported that their participation had allowed them to save time and had improved quality of care.

***Profile 3 active GPs ***had work habits that were not as well suited to the SIPA model. They had fewer SIPA patients in their practices (4.1 per physician on average). They were afraid that SIPA would consume too much of their time. They were satisfied with the physicians' recruitment and with their relationships with the case managers. Their mode of contact with the geriatrician was problem-free. They felt that they had not received enough information on their patients. They reported that SIPA was efficient and had provided quality services. They considered the physician-patient relationships as satisfying and the funding as appropriate compensation for the changes to their practices. Their working conditions were improved by the anticipation of patients' problems.

***Profile 2 non-active GPs ***had work habits that were similar to those of Profile 3, but they had fewer SIPA patients in their clienteles (2.4 per physician on average). They wanted support in providing care to their frail elderly patients. They more harshly criticized some aspects of the SIPA implementation, especially how physicians were recruited and the lack of information. They had little contact with case managers, who sometimes built exclusive dyads with the geriatrician. Then They disliked sharing clinical responsibility with the geriatrician. They were satisfied with the quality of SIPA services. They considered the funding useless and they reported having "lost" some patients during the project.

***Profile 4 non-active GPs ***had work habits that differed greatly from those required under the SIPA model. They had the fewest number of SIPA patients in their clienteles (1.1 per physician on average). They wanted to transfer their frail patients to SIPA and would have preferred to participate in the selection process in order to be able to transfer additional elderly patients to SIPA. They were rarely in contact with the case managers and were not in contact with the geriatrician. They felt that SIPA provided good quality services. They viewed the SIPA funding as a bonus. SIPA allowed them to save time in their practice, mainly because they were relieved of patients with more complex problems.

It thus appears that professional factors dominate in Profile 1 (active physician) and Profile 4 (non-active physician), where they are given more weight than organizational and financial factors. Profile 1 physicians had clinical practices that were compatible with the SIPA model, as opposed to Profile 4. The profile 1 GPs made more positive assessments of the SIPA model and participated much more actively. Profile 2 and profile 3 physicians have similar professional characteristics, and their types of practice differed significantly from SIPA. Their level of participation depended mainly on organizational factors. Profile 3 physicians had more SIPA patients in their clienteles, were more satisfied by the IHNS implementation (physician recruitment and the information provided), and were contacted more by the case managers during the project.

## Discussion

This study has provided an in-depth look at the incentives and barriers for securing GP active participation in IHSNs. Our study has shown that the participation of GPs in an IHSN is a complex phenomenon that depends upon a multitude of professional and organizational factors throughout the program implementation process. It has revealed that a GP's intention to participate is not necessarily synonymous with ongoing participation. Participation profiles were identified, revealing that active GP participation depends mainly on collaborative practices, the number of IHSN patients, having more concerns than expectations, close relationships with the case managers and the perceived efficacy of SIPA. Improved quality of care and positive impacts on professional practices were acknowledged by both active and non-active GPs.

Clinician characteristics appeared to play a critical role in IHSN participation. GPs with more elderly patients in their clientele were often more active in SIPA. They reported knowing the difficulties inherent in managing this population, a practice characterized by medical complexity and chronic health conditions, and interdisciplinary coordination[24]. As in other recent studies, general practitioners were found to participate more than specialists[25,26]. But the number of years of practice was the same for active and non-active GPs. This differs from what has been reported in previous studies, where GPs with fewer years of practice were found to participate more[27,28]. Our sample was representative of our general Montreal GP population characterized by long-term practices and few young physicians. Physician-patient relationships appeared to be a strong factor favoring participation. Active GPs expected to retain patients and non-active GPs reported having lost some patients to the case manager/SIPA-geriatrician dyad. Previous studies have highlighted the importance of the physician-patient relationship in improving the GP's as well as the patient's participation in integrated health service networks[29,30].

The SIPA geriatrician was acting as a consultant to the GPs. His role in the project was to support GPs and not to compete with them. Previous studies have shown that a prominent factor in GP participation in IHSNs is the influence of clinical leaders, both at the start of, but also throughout the project[28,31]. Our data suggest that the quality of the GP/case manager relationship is central to the success of IHSNs, and a close collaborative relationship would appear to depend, in large part, on the number of patients selected for the IHSN. The more patients that a physician has in the network, the more frequent are his or her contacts with case managers, and the better the resulting professional relationships. This result validates previous research, which has shown that GPs need to have a minimum of patients before they will be motivated to change their practice patterns[17]. Also, most of the GPs wanted their role in the structure clarified and would have liked to receive more information earlier. As pointed out in previous research, communication between disciplines is crucial[32] and physicians need time to get to know their partners and establish the kind of connections that ultimately lead to trusting relationships[33,34]. Consequently, a relatively short duration might impair this process.

While previous studies have highlighted the role played by physicians' perceptions of quality of care as an incentive to participation[35,36] and the impact of their participation in IHSNs on their working conditions[37], we found that these factors were present for both active and non-active GPs. While perception of quality of care is important, this factor did not appear sufficient to ensure participation; it needed to be associated with other incentives. In terms of the consequences on professional practices, active GPs wanted to keep their frail patients as compared to non-active GPs and this factor was related to the perceived efficacy of SIPA. In terms of remuneration, our data suggest that financial incentives have a limited impact on the level of participation, although the availability of additional funds appears to be useful in those cases where physicians first agree to change their practices in exchange for extra remuneration. For the other participants, the desired effect would appear to be limited.

Despite its contributions, our study has some limitations. It examined an IHSN serving a given target population, but we believe that most of the results could be verified in other populations. Another issue is that of the 23-month period between the end of the experiment and our study. This may have introduced some memory biases, mainly among the physicians who were not active. A cross-validation of our results seems to indicate that this was not the case. Perceived consequences have been evaluated following the SIPA program and could be related to the degree of medical involvement closing the gap between the intent and the reality of the program. Also, because we ensured anonymity to our respondents and guaranteed them that we would not share their identity with the SIPA team, it became impossible to pair our results with those of SIPA concerning the patients' outcomes. Future research should focus on these health status impacts when comparing active and non-active GPs. Finally, the evaluation of participation was carried out independently by three independent professionals (2 case managers and 1 manager of care) at each site. Since the definition of active versus non-active participation was relatively broad, it is possible that this methodology produced false-positive results.

Despite these limitations, this is the first study to examine GP active and non-active participation in an IHSN for frail elderly patients, and the similarity in the results from two sites improves the external validity of our findings. Active participation of GPs seems to play a critical role in the success of IHSNs; a better understanding of the factors that facilitate or hinder their implementation thus becomes vital. Years ago, the reduced choices available to physicians under models such as the PACE model in the USA was already associated with limited patient registration, as patients refused to give up their regular GPs[38,39]. In reaction, recent IHSN implementations such as SIPA have included GPs in the multidisciplinary team to improve model acceptance rates among frail patients. The results from our study will therefore be useful to improving the level of participation of both patients and physicians.

## Conclusion

Our results provide practical guidelines for establishing future integrated models of care. They reveal the importance of identifying, from the outset, the GPs who are most likely to be accustomed to caring for the elderly. It thus seems essential to recruit a significant number of IHSN patients per physician in order to reinforce the relationship between the GP and the case manager. Then, it is important to ensure that GPs will be informed during the implementation and meet personally with the multidisciplinary team, including case managers and the geriatrician. The relationship between the patient and their GP should be preserved and the physician should be directly involved in the care of her or his patients. Thus GPs should be accompanied by accessible and available geriatricians who can respond to their requests, support them during the project and encourage them to collaborate with the case managers without taking over the GP's role. Finally, it demonstrates the need to allocate sufficient time so that these programs can be truly tested. By raising levels of medical participation in IHSNs, we can create the kind of organization that will foster integrated services and stand squarely in the way of the current trend toward fragmentation.

## Competing interests

The authors declare that they have no competing interests.

## Authors' contributions

MDS, HB and LL designed the study. HB was involved in the SIPA experiment. MDS, IV and LL developed the structured interviews. MDS conducted the interviews. MDS, IV and LL analyzed all the interviews. All authors read and approved the final manuscript.

## Appendix

The 2 sites were in Montreal.

Site 1 was a multiethnic, mainly French-speaking neighborhood in the north east of the Island of Montreal. The socio-economic context was a population deeply split between rich and poor; average income per inhabitant was above the Montreal average, yet 36% of the population was living below the low income cut-off. At this site, frail elderly people had been selected for SIPA mainly through the site's home care services and recruiting through public notices.

Site 2 was a multiethnic, mainly English-speaking population in the western part of the Island of Montreal. In some of its neighborhoods, over 50% of the inhabitants were immigrants, and the site counted 14% of all recent immigrants to the Montreal region. There were dramatic contrasts in socio-economic levels, with marked disparities between neighborhoods. At this site, SIPA had selected frail elderly people in the same manner through their home care services. The teams at both sites were composed of case managers (a nurse or social worker), community nurses, social workers, occupational therapists, physiotherapists, homemakers, a part-time geriatrician and managers of care. There was one case manager for 40 frail elderly patients. Site 2 had a part-time consulting pharmacist.

## Pre-publication history

The pre-publication history for this paper can be accessed here:


